# How public and private health insurance coverage mitigates catastrophic health expenditures in Republic of Korea

**DOI:** 10.1186/s12913-022-08405-4

**Published:** 2022-08-16

**Authors:** Hyun Woo Jung, Young Dae Kwon, Jin-Won Noh

**Affiliations:** 1 Department of Health Administration, Graduate School·BK21 Graduate program of developing glocal experts in health policy and management, Yonsei University, Wonju, Republic of Korea; 2 Department of Humanities and Social Medicine, College of Medicine and Catholic Institute for Healthcare Management, The Catholic University of Korea, Seoul, Republic of Korea; 3 Division of Health Administration, College of Software and Digital Healthcare Convergence, Yonsei University, 1, Yeonsedae-gil, Heungeop-myeon, Wonju-si, Gangwon-do 26493 Republic of Korea

**Keywords:** Private health insurance; National health insurance, Insurance coverage, Republic of Korea, Catastrophic health expenditure

## Abstract

**Background:**

The private health insurance (PHI) market in Republic of Korea has instituted indemnity insurance plans that provide partial reimbursements for some medical services or costs that are not covered by the National Health Insurance (NHI). To date, no study has estimated the extent to which PHI coverage lowers the economic burden of households’ access to health care. The current study aims to evaluate the design of Korea’s PHI system in terms of coverage using a catastrophic health expenditure (CHE) indicator and compare it with NHI.

**Methods:**

This study determined the difference between the number of households that were subscribed to PHI and those that received reimbursements from PHI. Additionally, it compared the effects of reduced CHE by NHI benefits with PHI reimbursements. Furthermore, it compared PHI reimbursements based on income class. Finally, it analyzed the contribution of NHI and PHI to CHE reduction through a two-part model with hierarchical regression.

**Results:**

The results indicated that of the 5644 households examined, 3769 subscribed to PHI, but only 246 households received reimbursements. Notably, NHI reduced CHE incidence by 15.17%, whereas PHI only reduced CHE by 1.22%. The NHI scheme indicated reduced inequality as it provided more benefits to the low-income class for their used medical services, whereas PHI paid more reimbursements to the high-income class. Accordingly, NHI coverage has protected households from CHE and improved equality to some extent; however, PHI coverage has had a relatively low effect on relieving CHE and has increased inequality.

**Conclusions:**

The indemnity health insurance plans of PHI companies in Korea only cover partial medical costs or services, and so, most patients do not receive reimbursements. Thus, Korea’s PHI system needs to improve to provide benefits to patients more generously and alleviate their financial burden.

## Background

Since the early 2000s, it has become a major trend that the health care systems of developed nations move to a mixed version of public and private insurance systems [1–3]. This is because compulsory social insurance for essential packages of health care services alone cannot satisfy all medical needs, and it is challenging for households to bear the burden of high medical costs for other non-essential health care services. Therefore, many countries with public health care systems have introduced supplementary private insurance, topping up any remaining services with copayments [4].

Republic of Korea (hereafter, Korea) has introduced a national health insurance (NHI) scheme that includes the compulsory coverage of 97% of the population, except those recipients of Medical Aid that protect the accessibility of care for the poor [5]. However, the benefit coverage of NHI is rather low, indicating that the proportion of out-of-pocket (OOP) payments, including copayments for services that have been insured and full payments for uncovered services, is approximately 32.2% of the health expenditure in 2018. This metric is relatively higher than those of Japan (13%), Germany (12.6%), the UK (15.9%), and France (10.2%) [6]. If OOP payments increase excessively, catastrophic consequences for households and the economy may ensue [7]. The World Health Organization (WHO) [8] states that if the ratio of OOP expenses to a household’s ability to pay exceeds a specific threshold, it is considered as “catastrophic health expenditures (CHE),” and this has been adopted as a measure of fairness in financial contribution indicators [8, 9]. Consequently, many studies on CHE have been conducted in Korea for more than a decade, and almost all of these studies have criticized the financial functioning of the Korean NHI scheme, which barely protects households from high OOP expenses [10–12].

The pricing system of the health care service market in Korea is based on a fee-for-service scheme and NHI is a third-party payer that covers some proportion of medical fees. There are many services in the medical market, most of which are “covered” services managed by NHI, and other “non-covered” services. Notably, NHI covers some proportion of medical costs for services that are covered according to the coinsurance rates, and the rest of the expenses become statutory copayments of patients [13]. At the same time, the patients must make full payments for services such as dental prosthetics, vision correction surgery, manual therapy, and other treatments or medicine based on new health technologies. These uncovered services may have clinical evidence for their treatment effects. However, the NHI does not pay for them due to low economic efficiency or the existence of other alternative medical services.

Moreover, private health insurance (PHI) in Korea covers OOP expenses, the sum of statutory copayments, and costs for uncovered services [10]. Notably, PHI mainly sells two types of insurance plans—fixed benefits and indemnity. The indemnity health insurance plan partially reimburses the patient’s OOP payments. The fixed benefit insurance plan pays the precontracted amount in consideration of medical expenses, loss of income, and other expenses in the case of death, disability, or a few critical illnesses. Some of them are combined with private pension plans. Therefore, the WHO does not regard the fixed benefit plan as a component of the health care system because it coverage is beyond that of the health coverage scheme [14].

Many Koreans additionally purchase PHI plans, a supplementary scheme covering services not covered by NHI. Although some variations exist, depending on research data, it has been reported that approximately 65–80% of households have PHI plans [10, 15, 16]. Moreover, PHI premiums have averaged US$ 184.9 per household with PHI per month, which is 2.1 times higher than NHI contributions (US$ 89.9 per month) [16]. Given this difference in premiums, it would be reasonable for households insured with PHI to be able to significantly reduce their OOP expenses. Furthermore, NHI benefits (benefit-in-cash and benefit-in-kind) are the amounts that NHI pays for medical services according to coinsurance rates; PHI reimbursements are a part of the OOP expenses reimbursed by PHI.

Previous studies have argued that poorly-designed PHI systems increase the prevalence of challenging issues like inequality, insuring only young and healthy people, and causing cost escalation [4, 17]. Such studies have also suggested that well-designed PHI systems can help households avoid the financial shock of large OOP expenditures when accessing health care. However, studies that analyze the extent to which PHI relieves households’ economic burden are rare. Previous studies related to PHI have mainly focused on the effects of PHI subscriptions and the increase in health care use, including adverse selections and moral hazard issues [10, 18–20] as well as care-seeking behavior [21, 22]. Most studies have shown that PHI increases health care use [23–25]. However, these studies have limitations because they analyze the effects of PHI subscriptions on health care use without considering the possibility of receiving reimbursements and the attendant amount. Additionally, unlike NHI, the indemnity plans of PHI pay only a portion of statutory copayments and make special reimbursement contracts for some expensive medical services that are uncovered by NHI. Furthermore, PHI’s claim process and exact condition of reimbursements are strict. Therefore, only a small percentage of the insured would receive reimbursements. In order to establish the hypothesis that PHI increases medical service or health care use, the possibility that PHI reimbursements significantly reduce the financial burden on consumers must first be determined. However, in the previous research, there has been no indication of the level of PHI coverage in Korea.

Furthermore, previous studies analyzed only the CHE incidence when estimating the economic burden of households due to medical expenses. However, this method had limitations. First, it was calculated only by using OOP expenses relative to household income; thus, the level of health insurance coverage could not be found. Second, as the unit of CHE incidence is the number of households, it was difficult to adequately measure the economic burden and insurance coverage level. Recently, Jung and Lee [26] developed methods by recalculating the incidence and positive gap of CHE to estimate the effectiveness of insurance in covering CHE. Therefore, this study applied the methods of Jung and Lee [26] and aimed to evaluate the design of the PHI schemes in terms of coverage by estimating how significantly the private indemnity health insurance mitigates CHE in Korea. Through this, we will compare the effects of Korea’s NHI and PHI in reducing CHE and suggest directions for enhancing the coverage and role of PHI.

## Methods

### Study design

First, we investigated whether there was a difference between the number of households subscribed to PHI and the number of households that received reimbursements. We expected the households that received reimbursements to be few. Next, we compared the effects of reduced CHE through NHI benefits with PHI reimbursements. Third, we compared NHI benefits and PHI reimbursements based on income class. It may be that the higher the income class, the higher the likelihood of the reimbursement rate. Next, this study aimed to analyze the contributions of NHI and PHI in reducing CHE through a two-part model using hierarchical regression. The expected result was that the contribution of NHI toward mitigating CHE may be substantial and would offset the sociodemographic differences. Conversely, the contribution of PHI may be insignificant.

### Data source and study population

The present research used data of 2017 from the Korea Health Panel Study (KHPS), conducted by the National Health Insurance Services and the Korea Institute for Health and Social Affairs. The KHPS is a representative data source for analyzing health care use and expenditures that is open to the public. First, the KHPS employs two-stage stratified random cluster sampling based on the Population and Housing Census, which covers the entire Korean population in the relevant year. Second, the data include various variables, such as individuals’ socioeconomic characteristics, health behavior, and other related aspects of health care use, including NHI benefits, statutory copayments, PHI reimbursements, hospital visits, length of stay, payment for uncovered services, and disease code. In addition, the KHPS uses health insurance data and receipt checks at the National Health Insurance Services to prevent loss of information and recall bias errors. The number of household samples from the 2017 KHPS dataset was 6392. We excluded 748 households that were surveyed for OOP expenses but not for total health care payments (THP; OOP expenses + NHI benefits). These cases may include health care use outside of the formal institutional health system, such as alternative therapies. This study was approved by the Institutional Review Board of Yonsei University (approval number: 1041849–202,108-SB-127-01).

### Measuring CHE and the effectiveness of insurance coverage

Traditionally, CHE is calculated as the ratio of OOP expenses to the income level of household units [7, 27]. If the ratio of OOP expenses/income is greater than or equal to a threshold Z, it is considered “catastrophic.” This can be expressed as:

1 $$E_i=1,\mathrm{If}\;\frac{\mathrm{OOP}}{{}^`\mathrm{Income}}\geq\mathrm Z;E_i=0,\mathrm{If}\;\frac{\mathrm{OOP}}{\mathrm{Income}}<\mathrm Z)$$

Wagstaff and van Doorslaer [7] suggested three approaches for estimating CHE: incidence, positive gap, and mean positive gap. First, the incidence of CHE (*H*_*cat*_) is the proportion of CHE-occurring households to the total number of households (N). This is calculated as:

2 $$H_{cat}=\frac1N{\textstyle\sum_{i=1}^N}E1_i$$

Second, the positive gap of CHE (*G*_*cat*_) indicates the height of the OOP expenses as a share of the income based on the total population. The height of OOP shares (*O*1_*i*_) is calculated as $\frac{\mathrm{OOP}}{\mathrm{Income}}-\mathrm{Z}$. The *G*_*cat*_ is given as:

3 $$\frac1N{\textstyle\sum_{i=1}^N}O1_i$$

However, the positive gap approach has limitations vis-à-vis estimating the economic burden of households with CHE, as it is based on the total population, including households with no health care use. Therefore, Wagstaff and van Doorslear [7] suggested another approach called the mean positive gap (*MG*_*cat*_). This is calculated as:

4 $${\textstyle\sum_{i=1}^N}O1_i/{\textstyle\sum_i^N}E1_i.$$

Recently, Jung and Lee [26] developed methods to estimate the effectiveness of insurance in covering CHE. This method uses the difference between THP and OOP expenses to estimate the extent to which health insurance benefit payments reduce CHE.

First, it adopts the same processes as in Eqs. 1–4, except that OOP is substituted with THP. That is:

5 $$E2_i=1,\mathrm{If}\;\frac{\mathrm{TMP}}{{}^`\mathrm{Income}}\geq\mathrm Z;E2_i=0,\mathrm{If}\;\frac{\mathrm{TMP}}{\mathrm{Income}}<\mathrm Z)$$

6 $$K_{cat}=\frac1N{\textstyle\sum_{i=1}^N}E2_i,$$

7 $$J_{cat}=\frac1N{\textstyle\sum_i^N}O2_i\;\left(O2_i=\frac{THP}{\mathrm{Income}}-\mathrm Z\right)$$

8 $${MJ}_{cat}={\textstyle\sum_{i=1}^N}O2_i/{\textstyle\sum_{i=1}^N}E2_i.$$

Subsequently, the effectiveness of NHI coverage in reducing the incidence of CHE (*SH*_*cat*_) was calculated by *K*_*cat*_ − *H*_*cat*_, and the positive gap of CHE (*TS*_*cat*_) was calculated by *J*_*cat*_ − *G*_*cat*_. The mean positive gap of *TS*_*cat*_, *MTS*_*cat*_ is ($\sum_{i=1}^NO{2}_i-\sum_{i=1}^NO{1}_i\Big)/\sum_{i=1}^NE{2}_i$ (for a more detailed explanation, refer to Jung and Lee [26]).

To estimate the effectiveness of PHI coverage in reducing CHE, we used private health care payments (PHP; OOP expenses + PHI reimbursements). We define PHI benefit payments as the indemnity PHI products only because the WHO does not count flat-rate insurance as a component of the health care system [14].

9 $$E{3}_i=1,\mathrm{If}\ \frac{\mathrm{PHP}}{{}^{`}\mathrm{Income}}\ge \mathrm{Z};E{3}_i=0,\mathrm{If}\ \frac{\mathrm{PHP}}{\mathrm{Income}}<\mathrm{Z}$$

10 $$P_{cat}=\frac1N{\textstyle\sum_{i=1}^N}E3_i,$$

11 $${Sil}_{cat}=\frac1N{\textstyle\sum_{i=1}^N}O3_i\;\left(O3_i=\frac{THP}{\mathrm{Income}}-\mathrm Z\right)$$

12 $${MSil}_{cat}={\textstyle\sum_{i=1}^n}O3_i/{\textstyle\sum_{i=1}^N}E3_i.$$

Similarly, the effectiveness of PHI coverage in reducing the incidence of CHE (*SP*_*cat*_) was calculated using *P*_*cat*_ − *H*_*cat*_, and the positive gap of CHE (*TP*_*cat*_) was calculated using *Sil*_*cat*_ − *G*_*cat*_. The mean positive gap of *TS*_*cat*_, *MTP*_*cat*_ was computed as $\left(\sum_{i=1}^NO{3}_i-\sum_{i=1}^NO{1}_i\right)/\sum_{i=1}^NE{3}_i$.

### Statistical analysis

#### Descriptive statistics

Regarding the descriptive statistics, the characteristics of all the study subjects were shown using frequency and mean tests. All the study subjects were NHI subscribers because NHI subscription is compulsory for all citizens in Korea. To understand the PHI subscription and reimbursement rates, we separately presented households with PHI and households who received reimbursements from PHI, checking whether they differed according to the characteristics of the subjects through a chi-square test. We graphed the level at which NHI benefits reduced CHE and that at which PHI reimbursements reduced CHE.

#### Two-part model

When the dependent variable does not show a normal distribution and when the lower bound, usually 0, occupies a larger portion of the sample, the two-part model is an alternative method that overcomes the limitations of non-normality by dividing a single regression equation into two parts and analyzing it [28]. The first part is a logit or probit model that analyzes the effects of factors, such as the use of health care services. The second part is an ordinary least squares regression model, which estimates the effects of factors on the amount of health care use in those who embrace the health care system. The two-part model assumes that the determinants of health care use decisions and the amount of health care services are different. This model assumes that health care use decisions are mainly determined by predisposition factors, such as gender, marital status, and health status, and the amount is determined by economic factors, such as health insurance type or income level.

#### Hierarchical regression model

Hierarchical regression is a model that goes one step further from the multiple regression model that measures the relationship between various independent and dependent variables [29]. The hierarchical regression model considers a systematic order and hierarchy in the process of independent variables affecting the dependent variable. The method is simple. We can just add independent variables in the hierarchical order determined by the researcher to the existing regression model. In this process, the researcher can check the adjusted coefficient whenever a new independent variable is added and understand the relationship among the independent variables better as well as effectively comprehend the magnitude of the influence of a specific independent variable on the dependent variable [29]. The most significant advantage of the hierarchical regression model expands when new independent variables are discovered in the traditional model. The hierarchical regression can reveal the conventional model’s shortcomings and show how the model improves the precision of the estimation by adding new predictors.

#### Strategies for statistical analysis

We employed a two-part model to determine the factors of the incidence and positive gap of CHE (Model 1). Furthermore, to estimate and compare the effectiveness of NHI and PHI coverage, we applied hierarchical regression analysis to the two-part model. Model 2 adds NHI coverage (*O*2_*i*_ − *O*1_*i*_) to CHE in Model 1. Model 3 adds PHI coverage (*O*3_*i*_ − *O*1_*i*_) to CHE in Model 2.

##### Model 1

$$\mathrm{Part}\;1:\;\log{\left(\frac P{1-P}\right)}_i=\beta_0+\beta_1X_{1i}+\beta_2X_{2i}+\beta_3X_{3i}+\in_i$$

$$\mathrm{Part}\;2:\;log(Y\vert\:y\:>\:0)i\:=\:\beta_0\:+\:\beta_1X_{1i}\:+\:\beta_2X_{2i}\:+\:\beta_3X_{3i}\:+\in_i$$

P: *E*2_*i*_ (threshold: 10%), Y: *O*2_*i*_ (threshold: 10%), *X*_1*i*_: predisposing factors (gender, age, educational level, marital status, job type of household head), *X*_2*i*_: needs factors (with or without disabled, number of chronic diseases, and the experience of health care use of four major diseases (cancers, cerebrovascular diseases, cardiac diseases, rare diseases), *X*_3*i*_: enabling factors (income adjusted by household equalization index (*number of adults* + 0.5 × *number of children*)^0.56^ [8], with or without PHI, type of NHI, and region), and *ϵ*_*i*_: error term.

We used the incidence of CHE based on THP and *E*2_*i*_ as the dependent variable for the logistic regression in the two-part model and used the positive gap of CHE based on THP, *O*2_*i*_ for the linear regression in the two-part model. In the case of *O*2_*i*_, we applied a logarithmic transformation. Regarding the four major diseases, the NHI of Korea has increased the coverage rate as a special plan owing to high mortality and medical expenses. In Model 2, we added NHI coverage to CHE (*O*2_*i*_ − *O*1_*i*_) to Model 1, and in Model 3, we added PHI coverage to CHE (*O*3_*i*_ − *O*1_*i*_) to Model 2. We used the statistical software program Stata/SE version 14.0 (Stata Corp., Texas, USA) for all the analyses.

## Results

### General characteristics of the samples

By examining the general characteristics, 3769 out of 5644 households had PHI, and 246 households received PHI benefits. We described relationships between paid benefits and other characteristics in households with PHI (Table 1).

**Table 1:** General characteristics of samples

Variables			N (%)		
			Total	Insured in private health insurance	Paid benefits
Characteristics of householders	Gender	Men	4305 (76.28)	3051 (80.95)	193 (78.46)
		Women	1339 (23.72)	718 (19.05)	53 (21.54)
	Age	< 29	187 (3.31)	90 (2.39)	13 (5.28)
		30 ~ 39	676 (11.98)	454 (12.05)	52 (21.14)
		40 ~ 49	1166 (20.66)	761 (20.19)	83 (33.74)
		50 ~ 64	1727 (30.6)	1013 (26.88)	93 (37.80)
		> 65	1888 (33.45)	1451 (38.5)	5 (2.03)
	Education	Higher than college	1703 (30.17)	1123 (29.8)	114 (46.34)
		High school	2185 (38.71)	1487 (39.45)	92 (37.4)
		Less than middle school	1756 (31.11)	1159 (30.75)	40 (16.26)
	Marital status	Married	3929 (69.61)	2856 (75.78)	186 (75.61)
		Single	1715 (30.39)	913 (24.22)	60 (24.39)
	Job type	Employee	2430 (43.05)	1507 (39.98)	157 (63.82)
		Employer/self-Employed	1356 (24.03)	896 (23.77)	61 (24.80)
		Unemployed	1858 (32.92)	1366 (36.24)	28 (11.38)
Characteristics of households	Region	Urban	2244 (39.7)	1454 (38.6)	105 (42.7)
		Rural	3400 (60.3)	2315 (61.4)	141 (57.3)
	Income level	5th (rich)	1128 (19.99)	741 (19.66)	75 (30.49)
		4th	1128 (19.99)	724 (19.21)	61 (24.8)
		3rd	1129 (20.00)	695 (18.44)	71 (28.86)
		2nd	1129 (20.00)	760 (20.16)	33 (13.41)
		1st (poor)	1130 (20.00)	849 (22.53)	6 (2.44)
	Private health insurance	No	1875 (33.22)	–	–
		Yes	3769 (66.78)	3769 (66.78)	–
	Type of national health insurance	Employee	3731 (66.11)	2465 (65.40)	172 (69.92)
		Self-employed	1481 (26.24)	971 (25.76)	70 (28.46)
		Medical Aid	432 (7.65)	333 (8.84)	4 (1.63)
	Presence of disabled	No	5037 (89.25)	3322 (88.14)	235 (95.53)
		Yes	607 (10.75)	447 (11.86)	11 (4.47)
	Presence of four major diseases	No	4259 (75.46)	2762 (73.28)	210 (85.37)
		Yes	1385 (24.54)	1007 (26.72)	36 (14.63)
	Number. of chronic diseases (mean, S.D.)		0.567, 0.678	0.610, 0.701	0.610, 0.701
Number of samples			5644	3769	246

### NHI benefits, OOP payments, and PHI reimbursements by income quintile

There were differences in NHI benefits and PHI reimbursements according to income class. First, regarding NHI benefits, the average of the poor and the near-poor was the highest, decreasing for the higher income group. Second, there were few households who received reimbursements from PHI in the lower class, and the number of beneficiary households increased with the income class. In addition, the higher the income group, the higher the PHI reimbursement average. Finally, as for OOP expenses, the average payment among the poor was slightly lower than that of other groups; however, the other groups showed similar results (Table 2).

**Table 2:** National health insurance benefits, out-of-pocket expenses, and private health insurance benefits by income quintile

Income quartile	National health insurance benefits (dollar)			Out-of-pocket expenses (dollar)			Reimbursement from private health insurance (dollar)		
	n	Mean	S.D.	n	Mean	S.D.	n	Mean	S.D.
1st (poor)	1096	2375.82	4541.33	1096	1209.17	1694.55	6	1351.0	1755.47
2nd	1113	2463.50	4362.19	1113	1627.60	2106.19	33	762.11	666.34
3rd	1106	1925.73	3407.04	1106	1566.22	1931.47	71	864.78	1827.95
4th	1098	1644.84	3044.78	1098	1657.88	2062.06	61	851.90	1696.51
5th (rich)	1103	1707.51	3559.46	1103	1710.57	2150.51	75	3643.41	9508.58
Total	5516	2024.12	3839.85	5516	1554.74	2003.41	246	1706.84	5545.17

Exchange rate: 1dollar = Korean Won 1146.9 (2021.08.10)

### The level of protection from CHE of households with NHI and PHI coverage

In the OOP-based CHE results that were derived based on the traditional CHE calculation method, when the threshold was 10%, *H*_*cat*_ and *G*_*cat*_ were the highest at 19.26 and 2.76%, respectively, as the threshold increased to 20 and 40%, respectively. In contrast, *MG*_*cat*_ increased with the threshold. The results of the THP-based CHE suggested by Jung and Lee [26] and the PHP-based CHE included in this study were higher than the OOP-based CHE, but there was a difference in the level. The results of NHI and PHI coverage on CHE are presented in the right tab. First, NHI reduced the incidence of CHE (*SH*_*cat*_) by 15.17% at a threshold of 10%, while it reduced the proportion of health care expenses to income of households with CHE (*MTS*_*cat*_) by 33.46% (23.46% + threshold 10%). In contrast, PHI reduced the incidence of CHE (*S*P_*cat*_) by 1.22% at a threshold of 10% as well as the proportion of health care expenses to income of households with CHE (*MT*P_*cat*_) by 11.39% (1.39 + threshold 10%) (Table 3).

**Table 3:** Summary results of incidence and the positive gap of catastrophic health expenditure based on out-of-pocket expenses, total healthcare payment, and private health care payments

		Based on out-of-pocket expenses			Based on total health care payment				National health insurance coverage			
Threshold		10%	20%	40%		10%	20%	40%		10%	20%	40%
Incidence measures	*H*_*cat*_	19.26%	8.03%	2.43%	*K*_*cat*_	34.43%	19.95%	9.53%	*SH*_*cat*_	15.17%	11.92%	7.10%
Intensity measures	*G*_*cat*_	2.76%	1.52%	0.62%	*J*_*cat*_	10.83%	8.23%	5.48%	*TS*_*cat*_	8.06%	6.70%	4.86%
	*MG*_*cat*_	14.39%	19.14	26.17%	*MJ*_*cat*_	31.51%	41.38%	57.84%	*MTS*_*cat*_	23.46%	33.71%	51.29%
		Based on out-of-pocket expenses			Based on private health care payment				Private health insurance coverage			
Incidence measures	*H*_*cat*_	19.26%	8.03%	2.43%	P_*cat*_	20.48%	8.65%	2.73%	*S*P_*cat*_	1.22%	0.62%	0.30%
Intensity measures	*G*_*cat*_	2.76%	1.52%	0.62%	*Sil*_*cat*_	3.05%	1.73%	0.73%	*T*P_*cat*_	0.28%	0.20%	0.11%
	*MPG*_*cat*_	14.39%	19.14%	26.17%	*MSil*_*cat*_	14.94%	20.15%	27.62%	*MT*P_*cat*_	1.39%	2.38%	4.30%

Note: *H*_*cat*_ is incidence, *G*_*cat*_ is positive gap, *MG*_*cat*_ is mean positive gap of catastrophic health expenditure using out-of-pocket payments; *K*_*cat*_ is incidence, *J*_*cat*_ is positive gap, *MJ*_*cat*_ is mean positive gap of catastrophic health expenditure using total healthcare payment rather than out-of-pocket payments; P_*cat*_ is incidence, *Sil*_*cat*_ is positive gap, *MSil*_*cat*_ is mean positive gap of catastrophic health expenditure using private healthcare payments; *SH*_*cat*_ is incidence, *TS*_*cat*_ is positive gap, *MTS*_*cat*_ is mean positive gap of national health insurance coverage, *S*P_*cat*_ is incidence, *T*P_*cat*_ is positive gap, *MT*P_*cat*_ is mean positive gap of private health insurance coverage

**Fig. 1:**
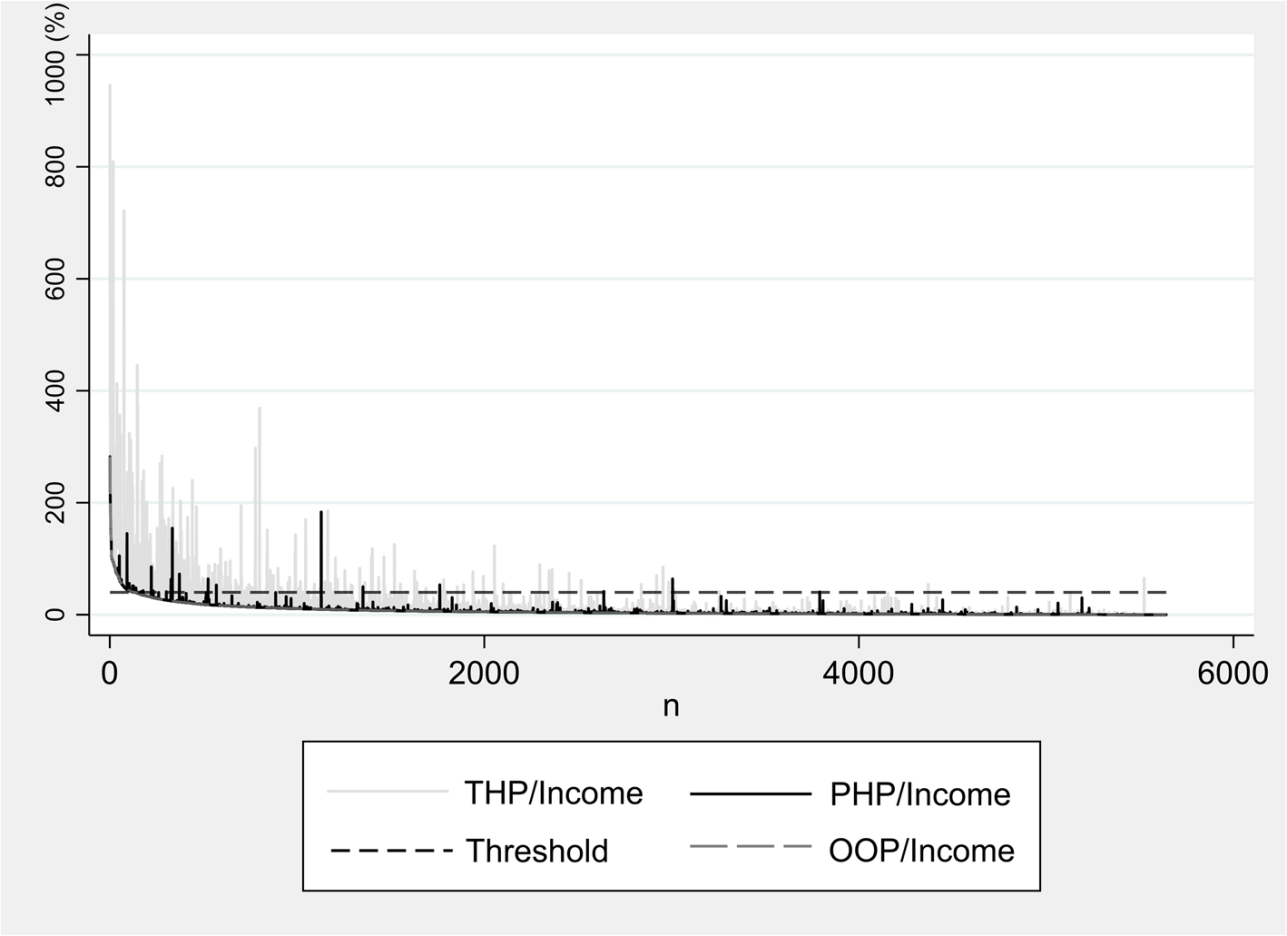
The role of national health insurance and private health insurance in protecting households from catastrophic health expenditure. The y-axis is the out-of-pocket payments as a share of the household’s income. The x-axis is arranged from left to right in the order of households with the highest out-of-pocket-to-income ratio. The red and blue bar graph represents the private healthcare payment (out-of-pocket + private health insurance benefit) and the total healthcare payment (out-of-pocket + national health insurance benefit) share of the household’s income. The smooth curve is the households’ out-of-pocket income

Figure 1 is a composite graph that visually shows the extent to which each NHI and PHI reduces CHE for each household. A smooth curve lying under the bars represents the ratio of OOP expenses to the income level of households in the order of highest to lowest. The heights of the blue and red bars represent the drop rates of CHE, which are covered by NHI and PHI, respectively. The bars fluctuate because they are all based on the ratio of OOP expenses to income. This means that someone may receive fewer NHI benefits, even if they pay higher OOP expenses. Evidently, compared to NHI coverage, PHI coverage was relatively infrequent and low in height. In addition, NHI coverage increased with the ratio of OOP expenses to income, whereas PHI coverage was irregular (Fig. 1).

### Coverage effects of NHI and private insurance on CHE

Model 1 is a typical factor analysis model for CHE (Table 4). Only the dependent variable CHE is based on THP and not the OOP expenses because this analysis aims to compare the impacts of the magnitude of NHI and PHI coverage. According to the results of the CHE incidence, logistic regression revealed that educational level, marital status, the household heads’ job type, household income level, type of NHI, presence (or absence) of the four major diseases, and the number of chronic diseases that affected CHE.

**Table 4:** Coverage effects of national health insurance and private health insurance on catastrophic health expenditure (hierarchical regression analysis)

		Model 1				Model 2				Model 3			
		Incidence		Positive gap		Incidence		Positive gap		Incidence		Positive gap	
		OR	S.E.	Coeff.	S.E.	OR	S.E.	Coeff.	S.E.	OR	S.E.	Coeff.	S.E.
Private Health Insurance coverage (*O*3_*i*_ − *O*1_*i*_)		–	–	–	–	–	–	–	–	1.458^*^	0.249	0.068^**^	0.022
National Health Insurance coverage (*O*2_*i*_ − *O*1_*i*_)		–	–	–	–	16.791^***^	1.768	0.936^***^	0.009	16.773^***^	1.774	0.933^***^	0.009
Gender (Male)	Female	1.015	0.138	−0.059	0.115	1.061	0.232	0.041	0.045	1.051	0.230	0.035	0.045
Age (< 29)	30–39	0.612	0.159	0.123	0.286	0.837	0.312	−0.088	0.113	0.826	0.308	−0.088	0.113
	40–49	0.756	0.184	0.132	0.260	1.024	0.359	0.008	0.102	1.009	0.354	0.004	0.102
	50–64	0.994	0.243	0.039	0.256	1.413	0.498	0.014	0.101	1.409	0.497	0.024	0.101
	> 65	1.237	0.314	0.109	0.262	1.519	0.566	0.003	0.103	1.520	0.567	0.017	0.103
Education level	High school	1.484^***^	0.166	0.208^*^	0.101	1.041	0.175	0.007	0.040	1.039	0.174	0.010	0.042
(> college)	< middle school	1.088	0.111	0.109	0.100	0.917	0.134	0.001	0.039	0.920	0.135	0.001	0.039
Marital status (married)	Single	0.632^***^	0.083	−0.017	0.111	0.734	0.154	−0.076	0.043	0.734	0.154	−0.075	0.044
Job type (employee)	Employer/self-employed	1.194	0.119	0.204^*^	0.088	1.039	0.156	0.006	0.035	1.034	0.155	0.002	0.035
	Unemployed	1.457^***^	0.142	0.290^***^	0.082	1.157	0.175	0.017	0.032	1.153	0.174	0.016	0.032
Region (Urban)	Rural	1.064	0.077	0.054	0.059	0.875	0.094	−0.011	0.023	0.879	0.094	−0.012	0.023
Income level (rich)*	Quintile 4	2.033^***^	0.298	0.014	0.171	1.872^***^	0.379	0.115	0.067	1.898^**^	0.385	0.127	0.067
	Quintile 3	4.184^***^	0.585	0.134	0.159	3.208^***^	0.631	0.102	0.063	3.249^***^	0.641	0.115	0.063
	Quintile 2	9.287^***^	1.328	0.523^***^	0.157	4.751^***^	0.986	0.205^***^	0.062	4.809^***^	1.000	0.215^***^	0.062
	Quintile 1 (poor)	17.002^***^	2.732	0.843^***^	0.163	6.472^***^	1.581	0.284^***^	0.064	6.564^***^	1.606	0.294^***^	0.064
Private health insurance (no)	Yes	1.161	0.104	−0.054	0.067	1.100	0.161	0.026	0.026	1.096	0.160	0.022	0.026
Type of national health insurance (employee)	Self-employed	0.936	0.077	−0.118	0.067	0.933	0.118	0.004	0.026	0.935	0.119	0.003	0.026
	Medical Aid	0.645^***^	0.081	0.073	0.093	0.630^*^	0.140	−0.188^***^	0.037	0.634	0.141	−0.186^***^	0.037
Disabled (no)	Yes	1.004	0.105	0.117	0.077	0.871	0.156	−0.017	0.030	0.869	0.156	−0.016	0.030
Four major diseases (no)	Yes	2.847^***^	0.225	0.386^***^	0.058	1.608^***^	0.202	0.001	0.023	1.591^***^	0.200	−0.001	0.023
Number of chronic diseases		1.618^***^	0.121	0.060	0.065	1.354^**^	0.158	−0.012	0.025	1.365^**^	0.160	−0.008	0.025
Constants		0.045^***^	0.012	1.561^***^	0.282	0.022^***^	0.008	0.254^*^	0.112	0.226^***^	0.008	0.237^*^	0.112
Model fits		0.2708^***^		0.144^***^		0.6437^***^		0.866^***^		0.6443^***^		0.866^***^	

*p < .05; **p < .01; ***p < .001

Model 2 adds NHI coverage (*O*2_*i*_ − *O*1_*i*_), which is the drop rate of CHE and the NHI coverage (Table 4). Notably, NHI coverage had an effect of 16.743 odds ratio in the logistic regression and a coefficient of 0.936 in the linear regression. After computing NHI coverage (*O*2_*i*_ − *O*1_*i*_), many social variables became insignificant except for household income level, type of NHI, presence of the four major diseases, and the number of chronic diseases. Moreover, there were significant changes among the variables. Compared to Model 1, the difference between income groups (odds, coefficient) was significantly reduced, and the positive gap for Medical Aid recipients was changed to a statistically significant decrease (coefficient: −0.188, *p* < 0.001). In addition, the effect of the four major diseases on the positive gap of CHE becomes insignificant.

## Discussion

This study evaluated the coverage of PHI for households by applying a modified CHE calculation method and compared it with the NHI coverage in Korea. A total of 3769 out of 5644 households subscribed to the indemnity plans of PHI, and only 246 households received PHI reimbursements. This revealed that NHI reduced health care inequality by providing more benefits to lower-income households. Conversely, the indemnity products of PHI provided reimbursements more to the higher-income households. This could be interpreted as an indication of the income-regressive aspect of PHI. In particular, the contribution of PHI to CHE reduction was relatively low compared to that of NHI in terms of incidence and positive gap indicators. The number and height of the bar graph in Fig. 1 show that the number of households with beneficiaries and the PHI reimbursements, which represents the effects of reduced CHE, is quite small compared to that of NHI.

The findings of the two-part model with the hierarchical analyses in Table 4 are presented as follows: Model 2 is the case whereby NHI coverage (*O*2_*i*_ − *O*1_*i*_) is added to Model 1. Moreover, NHI coverage had the most influence among all variables in the incidence and positive gap of CHE. When NHI coverage (*O*2_*i*_ − *O*1_*i*_) was added to Model 2, it offset the effects of other variables, which were significant in Model 1. Educational level, marital status, and job type were significant among the incidence of CHE in Model 1 but not in Model 2. This indicates that NHI effectively reduces the differences in health care expenses according to socioeconomic status. This interpretation can be validated by acknowledging that Korea operates a fee-for-service system, whereby NHI provides benefits according to the amount of health care used. In Model 2, the odds ratio and coefficient values of income decreased overall compared to those in Model 1 (Table 4). This can be interpreted as the maximum OOP expenses policy, which differentiates the burden of health care expenses according to income level, which has an effect to some extent. However, as the maximum OOP expense policy in Korea is only applied to the health care services covered by the NHI, excluding uncovered services, it seems that the difference in influence based on income level may not be completely offset.

Additionally, in the positive gap analysis, Medical Aid was not significant in Model 1 but decreased significantly in Model 2, and the presence of the four major diseases was significantly higher in Model 1 but not significant in Model 2 (Table 4). Medical Aid recipients in Korea pay only $1 or $2 as OOP expenses; thus, their health care expenses are very low compared to those covered by NHI. Therefore, the results of the positive gap, which appeared significantly negative (−), reflected reality more accurately. Second, Korea is implementing a policy (expansion coverage plan for the four major diseases) to lower the ratio of statutory OOP expenses to the total health care expenses to 5% for four specific diseases (cancers, cerebrovascular diseases, cardiac diseases, and rare diseases) that have high mortality and a high probability of causing high health care expenses [30–32]. Most Korean studies have concluded that the expansion coverage policy for these four major diseases is ineffective when analyzing CHE. However, we consider these results to be biased because the incidence rates do not change significantly. Studies that have analyzed the effects of policy on the four major diseases using OOP expenses or NHI benefits as a dependent variable tend to report that there is a policy effect [30, 33]; however, studies that adopt CHE incidence as a dependent variable tend to report no effect at all [34, 35]. In this regard, Jung and Lee [26] confirmed that the positive gap can be viewed more accurately than the incidence approach when considering policy effects. Overall, the fact that the four major diseases did not appear to be significant in Model 2 could be understood as the lowering of medical cost burdens by NHI.

The changes between Model 1 to Model 2 were dramatic, but not so in Model 3, which added PHI coverage (*O*3_*i*_ − *O*1_*i*_). Nonetheless, there are four significant results. First, all the regression coefficients for income in Model 3 were larger than those in Model 2, which indicated increasing inequality. In addition, the odds ratio of the four major diseases decreased from Model 2 to Model 3. This decrease can be interpreted to mean that the PHI coverage effect for these diseases exists because they are the covered under the main plans offered by PHI. In addition, the odds ratio of the number of chronic diseases was higher in Model 3 than in Model 2. This can be interpreted to be based on PHI’s non-acceptance of a high-risk group that may have many chronic diseases. Finally, the most important result was that PHI did not significantly contribute to the reduction of CHE. The reasons for the low coverage of PHI seem to be that indemnity insurance plans reimburse only for a small portion of statutory copayments and some uncovered medical services as well as the strict PHI claim process and detailed conditions of reimbursements.

According to the results of this study, most households were subscribed to PHI and paid premiums that were approximately twice that of NHI; however, the level of PHI coverage was rather low. The purpose of supplementary PHI was not only to meet the diversity of medical service demands but also to supplement the limitations of NHI coverage. Given that PHI in Korea is a part of the wider health insurance system, it cannot avoid the responsibility of protecting households. Therefore, it is necessary to enhance the coverage of indemnity insurance. To do so, the following issues should be addressed.

First, private insurance companies need to disclose transparent data. For example, PHI companies in Korea provide only part of their financial information to several PHI associations, and these PHI associations analyze the financial data and report them on the news. However, there is a lack of trust in such public domain disclosures because there are possibilities that the companies may hide certain information. The data on PHI used in this study were also collected from the patients rather than provided by private insurance companies. In particular, the major PHI companies in Korea announced in 2021 that they would significantly increase insurance premiums due to fiscal deficits. However, it is unknown whether the deficit is due to a large amount of reimbursements being paid to subscribers. In fact, it is possible that insurance plan sales performance is reduced because of the COVID-19 social distancing measures, which limit sales conducted by home salespersons, or that insurance cancellation increases due to the subscribers’ financial deterioration during the COVID-19 pandemic.

Second, it is necessary to establish a unified management system for mutual adjustments between NHI and PHI. For instance, in Ireland, PHI is managed by the Health Insurance Authority under the Ministry of Health and Central Bank of Ireland; hence, it is possible to understand and respond to the insurance market accurately [36]. However, in Korea, PHI is managed by the Ministry of Economy and Finance, and the Ministry of Health and Welfare governs NHI. Under this segmented system, managing the PHI market effectively is demanding. Even if an issue of insurance premium increase emerges, it would be impossible to duly determine whether there has been collusion among the PHI companies.

Third, there is no risk equalization scheme in Korea. It had been argued that the PHI plans made in the early 2000s were designed with generous coverage (combined with pensions), thereby causing deterioration of finance for private insurers over time. Now, albeit those plans have expired, the people who have subscribed since the 2000s have gradually become older and started to use more health care services, hence creating financial deficits for PHI. In Ireland, the Health Insurance Authority operates the “Risk Equalization Fund” [36]. This fund, raised through taxes from all the PHI companies, compensates insurance companies with a higher risk from the elderly. Likewise, a risk equalization scheme could be an option for stabilizing the financial soundness of the PHI companies in Korea. In summary, a new approach is necessary to reestablish the protective role of PHI by simultaneously expanding the benefits and solving funding problems.

To date, the level of practical PHI coverage has been filled with knowledge gaps, and this study provides basic data that aim to fill that gap for the first time. Most studies claim that PHI increases health care use when only considering enrollment status [10, 21–23, 37]; therefore, the level of reimbursements has not been included in the analysis. As a result of estimating the level of PHI coverage in this study, the coverage was found to be insignificant and the use of health care services was not problematic. In addition, it is difficult to assume if this would affect the NHI fund.

This study presents several limitations. First, although PHI is based on individual subscriptions, CHE is calculated at the household level; therefore, the PHI effect is also calculated at the household level. Second, the level of PHI coverage was somewhat underestimated due to the exclusion of fixed benefit insurance, savings insurance, and other types of plans, which were not included in the WHO’s standards for medical insurance. Third, this study did not conduct a longitudinal analysis because the PHI enrollment rate did not change, and the analysis mainly focused on the comparisons of NHI and PHI coverage. Fourth, this study did not consider the endogeneity issues rooted in sophisticated behavioral health economic theories such as adverse selection, favorable selection, and cream skimming. Because this study focuses on comparing the coverage rates of PHI and NHI, it is unnecessary to ensure homogeneity between groups. If follow-up studies investigate whether NHI pays more than PHI, in cases where patients have the same condition, they must consider the endogeneity issues using statistical techniques such as propensity score matching.

## Conclusion

This study reveals that PHI barely protects households from CHE. For private insurance to play the role of medical coverage that subsidizes NHI and reduce the burden of medical expenses of households, it is necessary to improve the benefit coverage of indemnity insurance. The Korean private insurance system needs institutional reforms throughout Korea’s private insurance market structure. In this regard, we propose disclosing transparent data for PHI and implementing a unifying management system and a risk equalization scheme.

## Data Availability

The raw data supporting this study’s findings are publicly available from the Korea Health Panel Survey with a permission. However, data availability for processed data is limited because the free sharing of data between individuals is prohibited according to the bylaws of the Korea Health Panel Survey. The processed data are available upon reasonable request and with the National Health Insurance Services and the Korea Institute for Health and Social Affairs’ permission.

## Abbreviations

NHI: National Health Insurance
OOP: Out-of-Pocket
WHO: World Health Organization
CHE: Catastrophic Health Expenditures
PHI: Private Health Insurance
PHP: Private Healthcare Payments
KHPS: Korea Health Panel Study
